# Correction: Up-converting β-NaY_0.8_[Yb_0.18_Er_0.02_]F_4_ nanoparticles coated by superparamagnetic γ-Fe_2_O_3_ nanosatellites: elaboration, characterization and *in vitro* cytotoxicity

**DOI:** 10.1039/d6ra90049f

**Published:** 2026-05-20

**Authors:** M. Parvizian, W. Mnasri, M. Pleckaitis, V. Karabanovas, H. Khan, S. Nowak, S. Gam-Derouich, L. Ben Tahar, O. Sandre, R. Rotomskis, S. Ammar

**Affiliations:** a Université Paris Cité, ITODYS, CNRS UMR-7086 Paris 75205 France merah@u-paris.fr; b National Cancer Institute, Biomedical Physics Laboratory Vilnius 08406 Lithuania; c Université de Carthage, Faculté des Sciences de Bizerte, Laboratoire Synthèse et Structure des Nanomatériaux Zarzouna 7021 Tunisia; d Northern Border University, College of Science, Department of Chemistry 73213 Arar Saudi Arabia; e Univ. Bordeaux, CNRS, Bordeaux INP, UMR-5629, LCPO Pessac 33607 France

## Abstract

Correction for ‘Up-converting β-NaY_0.8_[Yb_0.18_Er_0.02_]F_4_ nanoparticles coated by superparamagnetic γ-Fe_2_O_3_ nanosatellites: elaboration, characterization and *in vitro* cytotoxicity’ by M. Parvizian *et al.*, *RSC Adv.*, 2024, **14**, 31486–31497, https://doi.org/10.1039/D4RA00909F.

The authors regret that the link to the raw data in the data availability statement does not work. The correct link to the raw data for this study is: https://entrepot.recherche.data.gouv.fr/dataset.xhtml?persistentId=doi:10.57745/63QROU.

The Royal Society of Chemistry apologises for these errors and any consequent inconvenience to authors and readers.

